# One Health Interactions of Chagas Disease Vectors, Canid Hosts, and Human Residents along the Texas-Mexico Border

**DOI:** 10.1371/journal.pntd.0005074

**Published:** 2016-11-10

**Authors:** Melissa N. Garcia, Sarah O’Day, Susan Fisher-Hoch, Rodion Gorchakov, Ramiro Patino, Teresa P. Feria Arroyo, Susan T. Laing, Job E. Lopez, Alexandra Ingber, Kathryn M. Jones, Kristy O. Murray

**Affiliations:** 1 Department of Pediatrics, Section of Pediatric Tropical Medicine, National School of Tropical Medicine, Baylor College of Medicine and Texas Children’s Hospital, Houston, Texas, United States of America; 2 The University of Texas Health Science Center at Houston, School of Public Health, Houston, Texas, United States of America; 3 The University of Texas Health Science Center, School of Public Health, Brownsville Regional Campus, Brownsville, Texas, United States of America; 4 The University of Texas Rio Grande Valley, Department of Biology, Edinburg, Texas, United States of America; 5 The University of Texas Health Science Center at Houston, McGovern Medical School, Houston, Texas, United States of America; 6 Emory University, Rollins School of Public Health, Atlanta, Georgia, United States of America; US Food and Drug Administration, UNITED STATES

## Abstract

**Background:**

Chagas disease (*Trypanosoma cruzi* infection) is the leading cause of non-ischemic dilated cardiomyopathy in Latin America. Texas, particularly the southern region, has compounding factors that could contribute to *T*. *cruzi* transmission; however, epidemiologic studies are lacking. The aim of this study was to ascertain the prevalence of *T*. *cruzi* in three different mammalian species (coyotes, stray domestic dogs, and humans) and vectors (*Triatoma* species) to understand the burden of Chagas disease among sylvatic, peridomestic, and domestic cycles.

**Methodology/Principal Findings:**

To determine prevalence of infection, we tested sera from coyotes, stray domestic dogs housed in public shelters, and residents participating in related research studies and found 8%, 3.8%, and 0.36% positive for *T*. *cruzi*, respectively. PCR was used to determine the prevalence of *T*. *cruzi* DNA in vectors collected in peridomestic locations in the region, with 56.5% testing positive for the parasite, further confirming risk of transmission in the region.

**Conclusions/Significance:**

Our findings contribute to the growing body of evidence for autochthonous Chagas disease transmission in south Texas. Considering this region has a population of 1.3 million, and up to 30% of *T*. *cruzi* infected individuals developing severe cardiac disease, it is imperative that we identify high risk groups for surveillance and treatment purposes.

## Author Summary

In this study, we contribute to the growing body of evidence for autochthonous Chagas disease transmission in south Texas along the US-Mexico border. We found that coyotes, shelter dogs, and vectors in this region demonstrated high infection rates of *T*. *cruzi*. Random sampling of residents also revealed a higher than expected disease burden that had previously been undiagnosed. With up to 30% of infected individuals developing potentially fatal cardiac disease, it is imperative that we identify and treat patients before irreversible clinical manifestations have occurred. Future prospective studies are necessary to elucidate and validate the disease burden in this area.

## Introduction

Chagas disease (*Trypanosoma cruzi* infection) can cause fatal cardiomyopathy in up to 30% of infected people [1]. Transmission to mammals occurs via vector, oral, congenital, and/or transfusion/transplantation routes [2]. The triatomine vector, or “kissing bug,” serves as the predominate mode of transmission, particularly in established sylvatic and/or domestic transmission cycles [3]. Over 100 different wildlife mammalian species are competent reservoirs of disease and have been implicated in propagation of sylvatic transmission cycles in nature [4]. Canines, in particular, are important components of peridomestic transmission, resulting in a bridge between sylvatic and domestic transmission cycles [5–7]. Finally, human infections can occur when vectors establish nests inside or near the home, and vectors feed on both humans and domesticated animals [7, 8].

Disease prevalence is highest in impoverished regions of endemic countries due to a plethora of societal factors, including substandard living conditions that result in increased exposure to vectors [9]. While the southern United States is not traditionally considered an endemic area, recent evidence has implicated the establishment of vector transmission cycles, particularly in Texas [10, 11]. Historical evidence of *T*. *cruzi* infected vectors and mammalian reservoirs date back to the early 1900s [12]. While the first documented locally acquired human case was published in Corpus Christi, Texas in 1955, the south Texas region, including the Rio Grande Valley, has been the subject of investigation by public health authorities dating back to the 1940s [12].

South Texas has compounding factors that could contribute to this area being a high-risk region for transmission. Within the state, sylvatic transmission cycles have been reported with seven different vector species and 27 sylvatic mammalian reservoirs [10]. The potential for sylvatic spillover to humans in this region has been implicated from increased outdoor exposure and interactions in rural environments [13]. In addition, colonias (primarily Hispanic communities) in this region of Texas have unprecedented poverty rates and living conditions that allow for easy access for vectors to enter and colonize homes, which might place residents at an increased risk of domestic transmission [5, 14]. Despite this compounding evidence of increased potential for Chagas disease in the region, epidemiologic assessments are lacking. The aim of our current assessment was to ascertain the prevalence of *T*. *cruzi* in three different mammalian species (coyotes, stray domestic dogs, and humans) and vectors *(Triatoma* species) to understand the disease burden attributable to Chagas disease among sylvatic, peridomestic, and domestic cycles.

## Methods

### Ethics Statements

Texas Department of State Health Services in the lower Rio Grande Valley originally collected terminal samples of coyote sera as part of their rabies control programs in 2005–2006, and secondary aliquots from these specimens were shared for *T*. *cruzi* testing for the purposes of this study. Canine sera collection and Chagas disease testing were approved by the University of Texas Health Science Center Animal Welfare Committee (AWC-07-147 and AWC-03-029). For the human seroprevalence aspects of our study, the original Cameron County Hispanic Cohort study was reviewed and approved by the University of Texas Health Science Center at Houston Committee for the Protection of Human Subjects (HSC-SPH-03-007B), and Chagas disease testing on coded samples was approved under Baylor College of Medicine Institutional Review Board (H-32192).

### Study Population

We conducted a retrospective analysis of previously collected sera from coyotes, stray domestic dogs housed in public shelters, and residents participating in related research studies. With regards to the coyote specimens, secondary aliquots from specimens noted above were shared by the Texas Department of State Health Services for *T*. *cruzi* testing. For domestic dog specimens, sera were collected in 2007 and 2009 from juvenile (less than 6 months of age and over 8 weeks of age based on tooth development) stray dogs housed in public shelters at one of two locations (Brownsville in Cameron County and Edinburg in Hidalgo County). The rationale for collecting samples from dogs under 6 months of age was to identify new, acute cases of infection so that incidence, as opposed to prevalence, could be determined. We purposefully excluded puppies under 8 weeks of age to eliminate issues related to the possible transfer of Chagas-positive maternal antibodies.

Investigators from the University of Texas Health Science Center at Houston, School of Public Health, Brownsville Regional Campus, collected sera from an established cohort living in Cameron County, TX. The participants were recruited from randomly selected households between 2005 and 2008 as a means of assessing the general health of residents along the US-Mexico border. Potential participants were not excluded based on race/ethnicity, with all race/ethnicities eligible for study inclusion. Data from the original health questionnaire and echocardiograms performed by the Cameron County Cohort (CCC) study were available for descriptive analysis [15].

From 2012 to 2013, we received 115 Triatomine insects that were collected in peridomestic areas by citizens across 6 counties in south Texas. Insect specimens were shipped, typically live, to The University of Texas Rio Grande Valley for further processing. PCR testing was performed in collaboration with Baylor College of Medicine Laboratory for Vector-Borne and Zoonotic Diseases.

### *Trypanosoma cruzi* Diagnostics

Serum samples were thawed and analyzed using Chagas Stat-Pak and DPP assays (Chembio Diagnostic Systems, Inc, Medford, NY). These rapid immunochromatographic assays test for antibodies against *T*. *cruzi*. These highly sensitive and specific assays were designed for feasibility in field-testing of both human and canine blood [6, 16–18]. Tests were examined visually and scored as negative or positive, following manufacturer’s directions. A positive sample was defined as being positive on both assays. Negative samples included those that were positive on only one diagnostic but negative on the second diagnostic. Any equivocal samples were retested for further clarification. Due to the samples being retrospectively tested without potential for prospective clinical intervention and the exploratory nature of the project, additional confirmation testing with alternate diagnostics was not performed.

For *T*. *cruzi* testing and taxonomic species identification of *Triatoma* insects, the posterior third of the insects’ abdomen was homogenized with a 5 mm stainless steel bead in AL buffer (Qiagen, Valencia, CA) in TissueLyser II (Retsch, Haan, Germany) for 3 min at 25 Hz. Following manufacturer’s instructions, DNA was then extracted using DNeasy Blood & Tissue kit (Qiagen, Valencia, CA). *T*. *cruzi* DNA detection and insect-specific mitochondrial 16S DNA for speciation were performed using PCR and sequencing as previously described [8, 19].

### Data Analysis

Descriptive statistics were used to identify prevalence infection rates with 95% confidence interval (CI) and stratified by pertinent variables. For domestic dogs, positive infection was translated to incidence since all dogs would have acquired infection in the first 6 months of life. Statistical analysis was performed using STATA v12 (College Station, TX). Spatial analysis was performed using MapInfo Professional v11.5 (Stamford, CT).

## Results

### Chagas Seroprevalence in Coyotes

**Fig 1:**
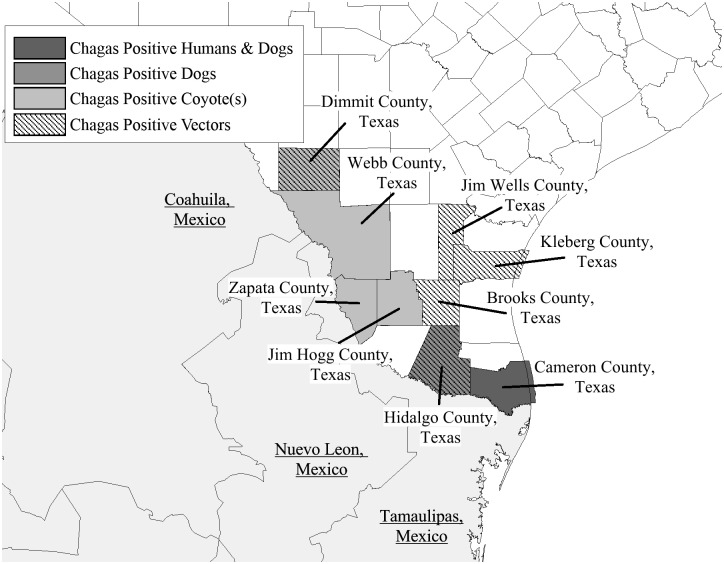
*Trypanosoma Cruzi* (Chagas Disease) Positive Samples By Species And Geographic Origin.

Coyote samples collected in the Rio Grande Valley had an overall seroprevalence rate of 8% (16 out of 199; 95% CI = 4.2% to 11.8%) (Table 1). Sampled coyotes were evenly distributed by gender (45% female) and all but one were adults. There was no difference in seropositivity by year of sampling. Interestingly, seroprevalence varied with regards to county of collection, with the highest seroprevalence identified in Zapata County (16%; 10/64), followed by Jim Hogg County (14%; 3/22), Dimmit County (10%; 2/20), and Webb County (1%; 1/83) (Fig 1). No positive coyotes were identified in Cameron, Hidalgo, Starr, or Wallacy counties, although sample sizes from each of these counties were low (range 1 to 4, total tested = 10).

**Table 1:** *Trypanosoma Cruzi* (Chagas Disease) Prevalence In Coyotes, Shelter Dogs, Human Residents, And Vectors Of South Texas.

Samples tested	Number tested	Chagas positive N (%)
Coyote (*Canis latrans*)	200	16 (8.0%)
Shelter dogs (*Canis lupus familiaris*) < 6 months of age	209	8 (3.8%)
Human adult cohort	841	3 (0.36%)
*Triatoma* species vectors	115	65 (56.5%)

### Chagas Seroprevalence in Domestic Canines

Samples collected from juvenile domestic dogs from neighboring Hidalgo and Cameron counties had an overall serologic incidence of 3.8% (8 out of 209 samples; 95% CI = 1.2% to 6.4%). We found a pronounced increase (4.4 fold) in Chagas incidence when comparing sampling in 2007 to 2009 (Fisher’s exact test, p-value = 0.04, 95% CI = 1.1 to 18.0), with 2% (3/152) of dogs positive in 2007 versus 9% (5/57) found positive in 2009.

### Chagas Seroprevalence and Clinical Data in South Texas Residents

Of 841 human sera samples tested from participants in the CCC, 3 individuals (0.4%; 95% CI = 0% to 0.8%) tested positive on both Stat-Pak and DPP assays. Limited residential history, medical histories and socioeconomic variables were reported as listed below. The precise origin and duration of their infection is unknown.

CCC Participant 1 was a 76-year-old female born in Canary, Texas (now known as Livingston, Texas) with a 52-year residential history in Brownsville, Texas. Case-patient 1 reported no current employment with an annual disability-benefit income of $3,336. Her medical history included diabetes, stroke, and hypertension. Case-patient 1’s mother was born in Texas while her father was born in central Mexico (Guanajuato). No data regarding any abnormal cardiac findings were available for this case-patient. On follow-up, participant’s husband reported that the participant had died recently with an apparent cause of death reported as leukemia.

CCC Participant 2 was a 45-year-old male born in San Luis Potosi, San Luis Potosi, Mexico with a 6-year residential history in Brownsville, Texas. In addition, he reported a prior 6-year residential history (while attending school) in the Brownsville, Texas border town of Matamoros, Tamaulipas, Mexico. Case-patient 2 was employed at the time of enrollment, reporting an annual income of $12,000. His past medical and social histories included diabetes and smoking. Both parents were born in north-central Mexico (San Luis Potosi). An echocardiogram performed on this participant showed normal left ventricular and right ventricular systolic function, mild concentric left ventricular hypertrophy, grade 1 left ventricular diastolic dysfunction, and no significant valvular abnormalities. The participant reported no symptoms related to any type of infection, and no additional cardiac evaluations were performed.

CCC Participant 3 was a 63-year-old male born in Matamoros, Tamaulipas, Mexico with a 22-year history of living in Brownsville, Texas. Case-patient 3 was retired with a prior occupational history in agriculture (occupational duration unknown) and a current annual income of $10,248. His medical history was negative for pre-existing conditions or co-morbidities. Case-patient 3’s parents were born in northern Mexico (Nuevo León). An echocardiogram performed at the same time as the original blood collection demonstrated normal biventricular systolic function, mild concentric left ventricular hypertrophy, grade 1 left ventricular diastolic dysfunction, and no significant valvular abnormalities. Similarly, the participant reported no symptoms, and no additional cardiac evaluations were performed.

### Prevalence of *T*. *cruzi* in Vectors

Finally, to determine the likelihood of infection in vectors in the region, PCR was performed on 115 insects (*Triatoma* species) collected around homes across 6 counties of south Texas. We found 65 (56.5%) positive for *T*. *cruzi* DNA, with prevalence ranked by county as follows: Brooks County (84%; 21/25), Hidalgo County (60%; 6/10), Jim Wells County (50%; 12/24), Kleberg County (47%; 22/47), Dimmit County (33%; 2/6), and Cameron County (0%; 0/1); 2 positive insects did not have a georeference provided. The most common insect collected was *Triatoma gerstaeckeri* (96.5% of insects; 62/111 *T*. *cruzi* positive), followed by *T*. *lecticularia* (2.6% of insects; 2/3 *T*. *cruzi* positive) and *T*. *sanguisuga* (0.9% of insects; 1/1 *T*. *cruzi* positive).

## Discussion

Chagas disease transmission has been identified along the Texas-Mexico border dating back to the 1970s [20, 21]. Our current study is the first to assess the infection status of vectors and seroprevalence among mammalian and human populations all living in the same geographic region of south Texas. Seroprevalence was highest among the sylvatic adult coyote reservoir (8%), moderate among peridomestic juvenile dogs in community shelters (3.8%), and lowest among local residents (0.36%), with one of the three positive CCC participants having a life-long history of living in Texas. In addition to finding evidence of infection in canines and humans, we found a high percentage (56.5%) of vectors carrying the parasite, further solidifying the risk of Chagas disease transmission in the region. Prior case reports have suggested the potential for domestic transmission along the eastern side of the Texas-Mexico border [5, 20], and now our larger regional assessment confirms this risk. Compounding evidence of poverty, substandard housing, rural residential exposure to sylvatic animals, and high infection prevalence of multiple species all can contribute to an increased risk of Chagas disease transmission to local residents [10, 14, 22].

Coyotes (*Canis latrans*) are den dwelling animals native to North America. Habitat preferences include caves and natural holes, or abandoned domestic structures such as drainage pipes, vacant homesteads and railroad tracks [23]. Similarly, triatomine vectors prefer natural or domestic habitats, living in large numbers within dens that provide constant access to a host meal source [3]. Our finding of 8% seroprevalence among coyote populations in the Rio Grande Valley is slightly lower than a prior study in 1978 which found a 12.8% (20 out of 156) prevalence of infection [20]. A second study published in 1984 found a 14% seroprevalence rate in coyotes from across Texas; however, none of the eastern Rio Grande Valley counties were included in this sampling [24]. Tennessee, Georgia, and Virginia are other southern states with known *T*. *cruzi* positive coyote populations [25–27]. Comparable to our study, these more recent studies found seroprevalence rates between 7–10%, suggesting that infection rates might be decreasing with time or current diagnostic tests have better sensitivity-specificity.

Dog (*Canis lupus familiaris*) populations in the United States can be feral or domesticated; however, both groups can serve as bridge hosts for transferring Chagas disease between sylvatic environments and humans. Dogs serve as important sentinel for disease surveillance purposes as their infection rates can be early predictors of transmission risk to humans, especially considering dogs develop clinical cardiac disease quicker than humans [5, 21, 28–30]. Using public health veterinary shelters as a sampling venue is a convenient methodology to capture feral, community-owned, and domesticated dog populations. The shelter dogs in our study of the Rio Grande Valley had a seroprevalence of 3.8%, which is considerably lower than other published infection prevalence estimates among shelter dog populations from across the state. Over 48 different dog breeds in Texas have demonstrated natural infection with *T*. *cruzi*, with prevalence estimates ranging from 8.8–20.3% [31, 32]. In the greater Brownsville, Texas area, infection prevalence of shelter dogs has ranged from 7.5% in 2003 to 6.7% in 2014 [5, 32]. While our prevalence is slightly lower than other studies, the reason is most likely related to our decision to sample dogs that were under 6 months of age, allowing us to estimate incidence related to recent vector-borne or congenitally-acquired infection. By estimating incidence, we can better understand the annual contribution of disease transmission in this geographic area.

The epidemiology and seroprevalence of human infection in the southern United States is largely unknown. Even in endemic areas, human seroprevalence is typically lower than sylvatic and domestic animals due to multiple factors, including increased mammalian-vector habitat exposure, mammalian predilection for oral ingestion of the triatomine vector, and varying defecation behaviors of different triatomine species [3, 30, 33]. While sylvatic transmission cycles between wildlife and vectors have been established in the southern United States, we are still in our infancy of understanding disease burden and transmission source in infected populations. A prior study conducted in 1977 found a seroprevalence of 2.4% (12 out of 500) among eastern Rio Grande Valley residents [20], which is a sharp contrast to our finding of 0.4% (3 out of 841). Our study sampling included random selection of participants, while their study biased their results by recruiting patients at Texas Chest Hospital in Harlingen. It is likely our sampling methodologies influenced the varying rates, especially as other historical random-selection population studies reported 0.01–0.9% seroprevalence [12]. Despite our selection methodology differences, both Burkholder et al.’s study and ours included long-time residents of the Rio Grande Valley, with one positive participant in our study very likely acquiring the infection in Texas. Based on our findings of a seroprevalence estimate of 0.4%, and considering a population of 1.3 million for the Rio Grande Valley, we can estimate that ~4,600 people in this region are currently infected with Chagas, with ~1,300 at risk for developing Chagas-related cardiomyopathy. If this estimate is accurate, then the burden of Chagas disease in the Rio Grande Valley is 23 times higher than what we had previously estimated based on our findings of 1 out of 6,500 (0.02%) blood donors in Texas testing positive for the disease [34]. Future studies should aim to further clarify the true disease burden and rate of autochthonous transmission in the Rio Grande Valley, an area with documented sylvatic and domestic *T*. *cruzi* transmission [5].

Our study had a few important limitations notable for discussion. The current World Health Organization guidelines require a minimum of two positive results on different antibody-based assays for diagnostic confirmation [35]. While we used two different assays, neither are currently FDA approved in the United States; however, Stat-Pak rapid immunochromatographic assay has demonstrated efficacy in all three populations of mammals in multiple studies [6, 16–18, 27]. For the purposes of this retrospective study we felt confident in the test results, especially as they were relatively consistent with other published literature. In addition to our finding of a high rate of infection (56.5%) among local vector species, other studies have also confirmed high rates of infection (51–82%) in Triatomine vectors throughout Texas [7, 8, 10]. Provided the retrospective nature of our study, the obvious lack of travel history in these coyote and dog populations, and the establishment of known *T*. *cruzi* positive vector populations in our study, we would argue that these are true infections acquired via local vector-sylvatic mammal transmission cycles. Another possible limitation, due to our retrospective sampling of frozen sera collected 8–10 years prior, is the potential for antibody decay resulting in a lower prevalence rate. Handling of the specimens included freezing aliquots to -80°C immediately following collection, constant monitoring of freezer temperature, and adhering to discipline standards during the serum thawing process in an effort to maintain sample preservation. Finally, we cannot rule-out the potential for cross-reaction with leishmaniasis. Rare reports of cutaneous leishmaniasis have been reported in the state [36]; however, none of our three Chagas-positive study participants presented with skin ulcers, lowering the potential for cross-reaction.

In conclusion, we contribute to the growing body of evidence for autochthonous Chagas disease transmission among mammals in south Texas. Coyotes, shelter dogs, and vectors in this region continue to demonstrate high infection rates of *T*. *cruzi*. Random sampling of residents also revealed a higher than expected disease burden that had previously been undiagnosed, with one human patient suspected of having locally acquired the disease. With up to 30% of infected individuals developing a potentially fatal cardiac disease, it is imperative that we identify and treat patients before irreversible clinical manifestations have occurred. Future prospective studies are necessary to elucidate and validate the disease burden in the Rio Grande Valley.

## Supporting Information

S1 Checklist Strobe Checklist. (PDF)
